# Co-Administration of Vadimezan and Recombinant Coagulase-NGR Inhibits Growth of Melanoma Tumor in Mice

**DOI:** 10.34172/apb.2021.037

**Published:** 2020-04-15

**Authors:** Amir Daei Farshchi Adli, Rana Jahanban-Esfahlan, Khaled Seidi, Davoud Farajzadeh, Ramezan Behzadi, Nosratollah Zarghami

**Affiliations:** ^1^Department of Medical Biotechnology, Faculty of Advanced Medical Sciences, Tabriz University of Medical Sciences, Tabriz, Iran.; ^2^Department of Cellular and Molecular Biology, Faculty of Biological Sciences, Azarbaijan Shahid Madani University, Tabriz, Iran.; ^3^North Research Center, Pasture Institute of Iran, Tehran, Amol, Iran.; ^4^Department of Clinical Biochemistry and Laboratory Medicine, Faculty of Medicine, Tabriz University of Medical Sciences, Tabriz, Iran.

**Keywords:** DMXAA, Tumor vascular infarction, B16-F10 melanoma cells, Cancer therapy

## Abstract

***Purpose:*** Tumor vascular targeting appeared as an appealing approach to fight cancer, though, the results from the clinical trials and drugs in the market were proved otherwise. The promise of anti-angiogenic therapy as the leading tumor vascular targeting strategy was negatively affected with the discovery that tumor vascularization can occur non-angiogenic mechanisms such as co-option. An additional strategy is induction of tumor vascular infarction and ischemia.

***Methods:*** Such that we used truncated coagulase (tCoa) coupled to tumor endothelial targeting moieties to produce tCoa-NGR fusion proteins. We showed that tCoa-NGR can bypass coagulation cascade to induce selective vascular thrombosis and infarction of mild and highly proliferative solid tumors in mice. Moreover, combination therapy can be used to improve the potential of cancer vascular targeting modalities. Herein, we report combination of tCoa-NGR with vascular disrupting agent (VDA), vadimezan.

***Results:*** Our results show that synergistic work of these two agents can significantly suppress growth of B16-F10 melanoma tumors in C57/BL6 mice.

***Conclusion:*** For the first time, we used the simultaneous benefits of two strategies for inducing thrombosis and destruction of tumor vasculature as spatial co-operation. The tCoa-NGR induce thrombosis which reduces blood flow in the peripheral tumor region. And combined with the action of DMXAA, which target inner tumor mass, growth and proliferation of melanoma tumors can be significantly suppressed.

## Introduction

Initially, the idea for targeting the tumor vascular system emerged as an interesting approach to treat cancer, however, the results from the clinical trials and drugs in the market were not much encouraging as it was expected.^1^ This is, the promise of anti-angiogenic therapy as the popular and well-practiced vascular targeting approach in the clinic was soon hindered by the development of drug resistance, disease relapse, and metastasis, knowing that tumors can apply redundant angiogenic signaling pathways. Although, they may take advantage of surrounding normal vascular systems through co-option mechanism.^2^

Tumor vascular infarction is turning into one of the most popular strategies for the treatment of solid tumors.^3^ That is, as tumor cells are fast-growing in nature they typically require a more efficient vascular system to meet the oxygen and nutrient demands of the high metabolically active cancer cells.^4,5^

Resembling cardiac infarction, in which a single clot in a vessel can implicate blood circulation to a large proportion of corresponding cells, for the first time this concept attempted by several groups using human tissue factor (TF) to induce vascular thrombosis in tumor feeding blood vessels to spark infarction and elicit subsequent tumor infarction.^6^ It follows that a successful blockade of a single tumor vessel would eradicate thousands of cells in the infarcted area.^7^ For targeted therapy of cancer and induction of tumor-specific thrombosis, truncated forms of TF (tTF), as the first member of vascular infracting agents, was used in fusion with different tumor endothelial targeting moieties for therapy of a myriad of different solid tumors in animal models.^1^ Small molecules such as NGR and RGD have made ideal homing peptides with selective affinity to integrin receptors frequently expressed on the surface of tumor endothelial cells.^8^ The tTF-NGR fusion protein currently is in phase I clinical trial.^9^ The beauty of tumor vascular infarction has been illustrated in two valuable papers that employed nano-drug delivery systems. In the first study, DNA nanotechnology using DNA nanorobots capable of smart opening at the tumor site to reveal hidden thrombin inside used for selective on-demand induction of tumor vascular infarction.^10^ Likewise, a two-component system is described where induction of coagulation cascade by either heated gold nanorods or tTF-RGD was exploited to broadcast the signals of coagulation to the receiving nanoparticles coated with fibrin binding peptide or FVIII substrate. Clot-targeting drug-loaded nanoparticles resulted in amplified anti-cancer chemotherapy up to 40 times compared to non-broadcasting nanoparticles.^11^ In another interesting study, pH-responsive Mg_2_Si nanoparticles are reported that can react and consume oxygen, forming a product that is capable of induction of thrombosis. This method is limited due to its systemic effects yet.^12^

With the aim of envisioning local effects, our group exploited unique coagulation properties of staphylocoagulase, which directly recruits prothrombin to trigger blood coagulation, in absence of inducing a cascade of clotting reactions, thus it avoids systemic effects of TF.^13^ Our results with truncated coagulase (tCoa) fused to RGD or NGR (tCoa-RGD/tCoa-NGR) demonstrated that they both were capable of significant induction of vascular thrombosis in the medium and high growth rate solid tumors in mice.^8,14^ Accordingly, beside systemic effects, inflammatory state and deep vein thrombosis in cancer patients should take into consideration upon administration of thrombotic agents.

Vascular disrupting agents (VDAs), that cause specific and irreversible destruction of the tumor, are one of the most promising members of tumor vascular targeting strategies. DMXAA is the main member of the VDAs family, which is currently undergoing extensive pre-clinical testing. DMXAA, also known as vadimezan or ASA404, a member of the flavonoids family, has been developed by researchers at the Auckland Cancer Society Research Centre.^15^ Proposed mechanisms for DMXAA function include direct effects such as induction of apoptosis in endothelial cells, resulting in complete vascular destruction, as well as hemorrhagic and ischemic necrosis in tumor tissue.^16^ Vascular degeneration is the first effect of DMXAA that occurs about 30 minutes after injection.^17,18^ Activation of the innate immune system is the indirect function of DMXAA, which is not seen with other VDA members.^19,20^ The production of inflammatory cytokines such as tumor necrosis factor (TNF), interleukin-6 (IL-6), or granulocyte colony stimulatory factor and several other chemokines such as IP-10 and monocyte chemotactic protein-1 are among DMXAA-related factors.^15,21^ Moreover, DMXAA activates NFκB signaling which is involved in the production of TNF and other cytokines.^22,23^ DMXAA also increases nitric oxide (NO) production.^24^ NO and TNF cause the deformation of endothelial cells in the vascular wall of the tumor.^17^

No specific molecular target for DMXAA has yet been identified in humans, but it seems that induction of the innate immune system in mice is done through human stimulator of interferon gene (STING).^25-27^ In fact, despite the promising result of DMXAA in the pre-clinical and phase I and II clinical studies, poor results have hampered the phase III clinical trials of the DMXAA, most likely because DMXAA is unable to activate the STING.^15^ Studies have been conducted to find the possible molecular target for DMXAA in humans or to construct an analog of DMXAA that could be a strong human STING activator.^28-31^

Another problem with VDAs is that they only kill about 90% of the tumor cells and 10% of the tumor cells in the peripheral region remain intact, which promotes further tumor relapse and metastasis and is hardly curable. These residual cells around the tumor, the rim effect, is the main problem with all VDAs.^32^

In this study, we intend to use the simultaneous benefits of two strategies for inducing thrombosis and the destruction of tumor vasculature as spatial co-operation.^33^ The induction of infarction by tCoa-NGR in the tumor reduces blood flow in the peripheral region, which can survive the thrombotic effects of DMXXA. In fact, we use two different mechanisms of action in the same place, so that we can achieve a higher activity at a lower dose.

## Materials and Methods

### Mice, cell line and agents

Mice (3- to 5-week-old, C57Bl/6 females) were obtained from the Pasteur Institute of Amol. Mice kept in a pathogen-free standard facility. Animals treated in accordance with the regulations of the Laboratory Animal Ethics Committee of Tabriz University of Medical Sciences (license NO: 93/3-4/3). Murine melanoma B16-F10 (ATCC) cultured in RPMI 1640 supplemented with 10% fetal bovine serum. Cell cultures maintained in a standard 37°C, 5% CO_2_ incubator. DMXAA purchased from Sigma Aldrich (catalog number D5817).

### Gene construction, cloning, expression, purification and characterization of tCoa and tCoa-NGR fusion proteins

All techniques performed in reference to our earlier work with tCoa-NGR.^14^ To this, coagulase gene sequence (Accession Number: KX914667.1) coding for full-length coagulase (~2 kbp) isolated from a native *Staphylococcus aureus* (ATCC 29213) was used for cloning of gene constructs consisting of truncated ~1.2 kbp form of coagulase gene (tCoa) fused to an NGR sequence (tCoa-NGR). Functional studies to determine fusion protein activity were performed according to our previous work.^14^

### Tumor mouse models

Murine melanoma B16-F10 cell lines grown to 70%–80% confluency. Then, C57/BL6 mice inoculated by subcutaneous (s.c) injection of 2×10^6^ melanoma cells in 3–5 week-old C57BL/6 mice. Tumor volume monitored and measured with caliper each day using the formula: volume (mm^3^) = width ^2^ × length × 0.5. Mice were randomly divided into four groups of 6 mice each, including group I: PBS, group II: tCoa-NGR, group III: DMXAA and group IV: tCoa-NGR+ DMXAA.

### DMXAA andtCoa-NGR treatment studies

Once tumors reached a volume of 200-300 mm^3^, mice injected with DMXAA (i.p) at the dose of 25 mg/kg body weight on the first day. The second and fourth days after the injection of DMXAA, two injections of the purified tCoa-NGR at thedose of 10µg diluted in 200 µl PBS administered intravenously. The same schedule used for single-agent therapies. The animals sacrificed on the 8^th^ day after the first treatment.

### Histology

To evaluate the overall efficacy of combination therapy of DMXAA and tCoa-NGR, tumor tissue collected, fixed at 10% buffered neutral formalin overnight, embedded in 2% paraffin, sectioned, and stained with H&E as described in our previous work.^14^

### Flow cytometry

In order to assess DMXXA-mediated tumor immune responses, tumor tissue was analyzed using flow cytometry (BD FACSCaliber). To this, mice sacrificed on the 8^th^ day after the first injection, and tissue samples were collected for obtaining a single-cell suspension. For tissue digestion, a digestion mix containing 1 mg/mL collagenase A, 0.4 mg/mL hyaluronidase type V and 0.04 mg/mL DNase I was prepared and used for digestion of 0.5 g of tumor tissue. The following antibodies used to identify the subpopulations of T lymphocytes, CD4-FITC, CD8-PE. Level of natural killer (NK) cells assessed by CD49b-FITC antibody.

### Evaluation of tumor cytokines level by ELISA

Intratumoral protein levels of the TNF-α and IL-6 measured in melanoma tumors using the enzyme-linked immunosorbent assay (ELISA) purchased from R&D systems.^34^ Tumor tissues were excised and homogenized in cell lysis buffer composed of 10 mm HEPES (pH 7.9), 1 mm EDTA, 60 mm KCl, 0.2% NP40, 1 mm DTT, and proteolytic enzymes. Supernatants were collected and analyzed for cytokine expression. A minimum of three mice used for each test group.

### Statistical analysis

In this study, data presented are mean ± standard error of the mean (SEM) of the three independent experiments. For statistical analysis, Kruskal-Wallis and multiple comparisons of mean ranks were used for all test groups. Differences in *P* values of 0.05 or less were considered significant.

## Results and Discussion

### Co-administration of tCoa-NGR fusion proteins and DMXAA reduces tumor growth in mice

**Figure 1:**
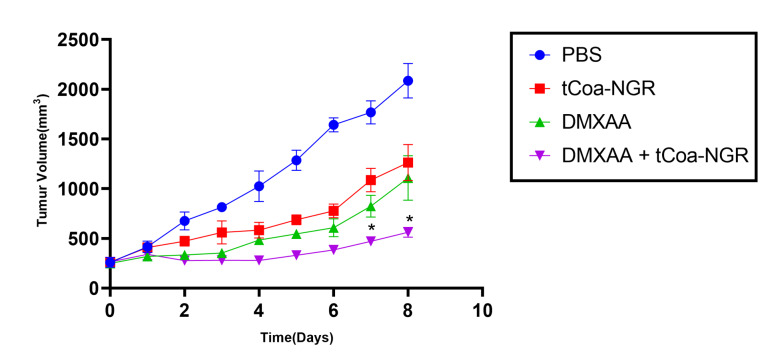
Treatment of mice bearing melanoma tumors with DMXAA and tCoa-NGR. DMXAA administered once (25 mg/kg) on the first day of the experiment and tCoa-NGR administered (10µg) two times at days 2th and 4th of the study. Inhibition of tumor growth was statistically significant after the administration of combination therapy (* *P* < 0.05).

Combined therapeutic effects oftCoa-NGR and DMXAA were evaluated in C57BL/6 mice bearing melanoma cancer. The single-dose intraperitoneal administration of DMXAA at the dose of 25 mg/kg alone reduced tumor volume compared with the PBS injection. Two injections of tCoa-NGR at the dose of 10µg with two days intervals on the second day after injection of DMXAA leads to a further decrease in tumor volume compared with DMXAA alone. In addition, tumor volume in DMXAA and tCoa-NGR groups were significantly reduced compared with the control group (PBS). On day 8 after the first injection, the volume of tumors in mice treated with a combination of DMXAA and tCoa-NGR was 73.12% smaller than the volume of control tumors. In DMXAA and tCoa-NGR groups, the tumor volume was 46.9% and 39.5% smaller than control tumors, respectively (Figure 1).

### Administration of DMXAA and tCoa-NGR increases damage in the tumor blood vessels

**Figure 2:**
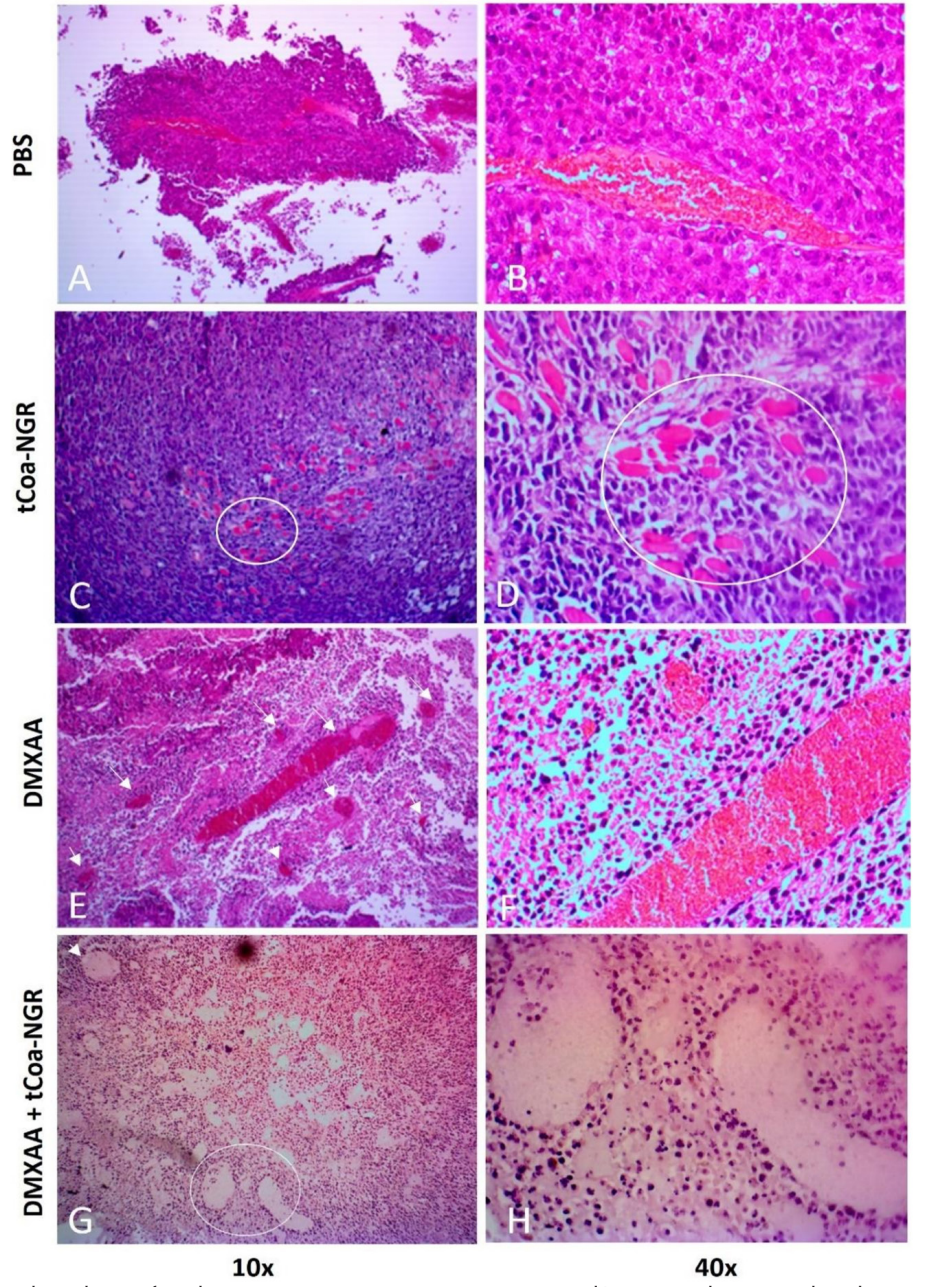
Histological analysis of melanoma tumor tissue sections in C57Bl/6 mice administered with PBS, tCoa-NGR, DMXAA, and combination of tCoa-NGR and DMXAA. **(A-B)** In PBS control group, blood vessels are intact, and tumor cells are alive. An example of a large blood vessel filled with intact red blood cells in highly-vascularized melanoma is shown (vessels are shown with arrows in A). **(C-D)** In group injected with tCoa-NGR, there are obvious signs of massive vascular thrombosis as indicated by a condense network of fibrin clot. An area with numerous occluded blood vessels is shown inside a circle. **(E-F)** DMXAA induce massive vascular damage at the tumor center. A large blood vessel filled with RBC is shown. **(G-H)** In combination group (tCoa-NGR+DMXAA) all blood vessels are damaged, and tumor cells are necrotic, degenerated and their structural integrity is lost. This potential is absent in single therapy modalities, indicating the suppressive action of dual targeting approach on the growth of highly aggressive B16F10 melanoma cancer cells in mice.

H&E staining used to assess and validate the effect of DMXAA and tCoa-NGR on reducing tumor volume in studied animals. Histological studies showed that the tCoa-NGR and DMXAA combination resulted in apparent vascular disruption, while tCoa-NGR leaves some vessels intact, and DMXAA only disrupts central vessels. The results indicate that a combination of DMXAA and tCoa-NGR could enhance the overall efficacy of cancer therapy (Figure 2).

### Administration of DMXAA increases the population of CD4^+^, CD8^+^ and NK immune cells in melanoma tumors

**Figure 3:**
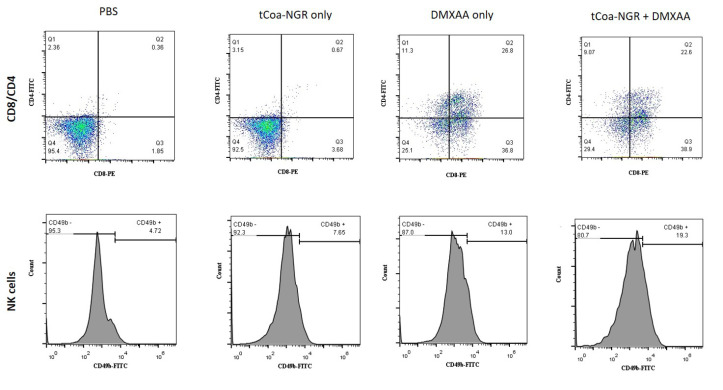
Increase of immune cells in melanoma tumor tissue after administration of DMXAA. DMXAA group and the combination of DMXAA with tCoa-NGR increased the levels of CD8 + lymphocytes, NK cells and to a lesser extent CD4 + lymphocytes. tCoa-NGR alone did not change populations of CD8^+^, CD4^+^, NK cells comparing to control (PBS).

DMXAA significantly increased the percentages of CD8^+^, CD4^+^, and NK cells in the DMXAA group and tCoa-NGR and DMXAA combination therapy group. Percentage of CD8^+^ cells increased from 1.85% in the PBS group to 36.8% in the DMXAA group, and to 3.68% in the tCoa-NGR group, and to 38.9% in the group that received the combination of DMXAA and tCoa-NGR. As expected tCoa-NGR did not increase the percentages of CD8^+^, CD4^+^, and NK cells significantly. Moreover, the percentage of CD4^+^ cells increased from 2.36% in the PBS group to 11.3% in the DMXAA group, and to 3.15% in the tCoa-NGR group, and to 9.7% in the group that received the combination of DMXAA and tCoa-NGR. In addition, the percentage of NK cells increased from 4.72% in the PBS group to 13.0% in the DMXAA group, and to 7.65% in the tCoa-NGR group, and to 19.3% in the group that received the combination of DMXAA and tCoa-NGR (Figure 3).

### Administration of DMXAA increases TNF-α and Il-6 levels in tumor tissue

**Figure 4:**
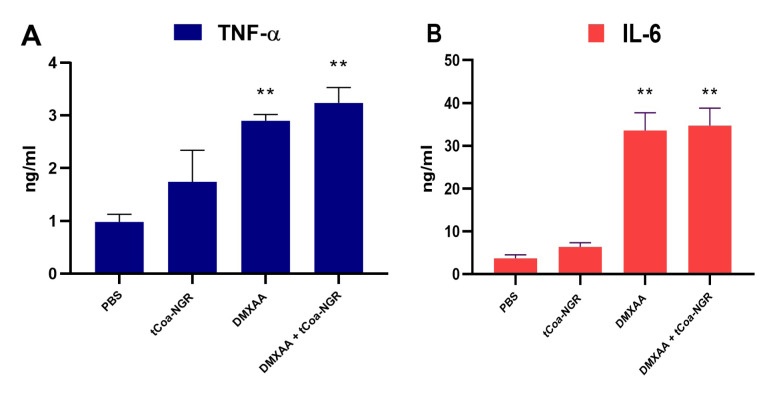
Cytokines change in different treatment groups. There was a significant increase in TNF-α (A) and IL-6 (B) levels upon administration of DMXAA and a combination of DMXAA and tCoa-NGR compared to PBS control. No significant changes in cytokine levels observed in tCoa-NGR group compared to the PBS control group (*P < 00.05).

Eight days after DMXAA injection, the amount of TNF-α and IL-6 were measured in tumor tissue sections. DMXAA significantly increased the level of TNF-α and IL-6, but the increase in TNF**-**α and IL-6 caused by tCoa-NGR injections were not significant. Also, DMXAA injection increased the TNF-α level in the tumor tissue by ~3.2 and 2.9 times, respectively (Figure 4).

Targeted combination therapy has gained momentum in solid tumor therapy in recent years.^35,36^ Tumor vessels are an excellent target for treatment because they play a vital role in survival and metastasis. Due to the complexity of the angiogenesis pathways and the tumor microenvironment, existing strategies such as anti-angiogenesis or vascular destruction factors have not been able to provide effective monotherapy treatment. About 75% of the clinical trials conducted on DMXAA are performed as a combination therapy since drug efficacy increases dramatically.^15^

Other vascular targeting methods, such as induction of infarction in the tumor, is an additional therapeutic option.^1^ As in our report, results indicated that a combination of the VDA (DMXAA) and clot-inducing factor (tCoa-NGR) by increasing the infiltration of innate immune cells in the tumor microenvironment and decreasing tumor volume could be a promising approach to achieve a higher therapeutic efficacy.

The tumor rim area is a major problem in many types of tumor-vascular based therapies. An area on the margin of the tumor that survives after treatment due to its proximity to normal cells and causes a recurrence of the tumor.^3^ In this line, the effect of DMXAA treatment in the peripheral region is as good as its function in the central parts of the tumor, but because of the proximity to the normal cells, tumor cells residing in the outer layers can survive DMXAA-mediated vascular disruption.^15^ To target both cells at once, one can disrupt the blood supply to the tumor periphery using another tactic to improve the treatment efficacy.^37^ Recently, thrombosis inducers have been used to treat solid tumors. Meanwhile, smart targeting of tumors by these agents through monoclonal antibodies^38,39^ peptide mediators such as NGR^40^ and RGD^41^ has created a high specificity in this therapeutic strategy.

The clot-inducing factors trigger blood coagulation through cascading events, resulting in ischemia and tumor cell destruction. The reduction or complete blockage of blood flow in all areas of the tumor that contains endothelial cells displaying surface αvβ3 integrin receptors as well as CD13 markers (ligands for NGR tripeptide), will occur.^42^ So it can be expected that combining tCoa-NGR with vascular damaging agents, such as DMXAA, can be more effective, as in our study and in line with our previous observations,^3^ the anti-tumor effects of tCoa-NGR start to vanish following cessation of treatment around day 5 of the study. A combination of DMXAA with tCoa-NGR, however, produced sustained thrombosis that followed by sustained tumor-suppressing potential by the end of the experiment. An additional benefit attributed to DMXAA, but not clot-inducing agents, is a direct or indirect disruption of tumor immune suppression by activation of the NFκB signaling pathway to promote anti-tumor immune response.^15^ Administration of a single dose of DMXAA, as a potent VDA, prior to induction of selective and local thrombosis by tCoa-NGR, resulted in reduced tumor growth rate. Thus, the promising anti-cancer effects suggest the efficacy of this synergistic formulation to shout down tumor vascular systems from the inside (DMXXA) and outside (tCoa-NGR).

Studying immune responses and vascular targeting effects at once requires an ideal tumor model. In this regard, we used the B16-F10 murine melanoma model for two reasons: first, highly proliferative and well-vascularized nature renders it suitable for vascular targeting therapeutics such as DMXAA. Also, the expression of CD13 surface markers can benefit melanoma from tCoa-NGR therapy.^43^ Second, as DMXAA treatment results in the infiltration of macrophages, immune cells, and cytokine release, thus, immune-related responses attributed to the effects of DMXAA can be studied in B16-F10 tumors, as an accepted model for immunotherapy studies.^44^

Combination therapy regimes consisting of DMXXA and other agents are also reported to achieve a synergetic and effective anti-cancer modality. Such that, Smolarczyk et al^37^ used the combination of DMXAA and digoxin to improve overall function. DMXAA only inhibits tumor for a limited time and space, so the purpose of this study is to use digoxin, which itself is a tumor suppressor, along with DMXAA to increase anti-tumor efficacy. The results of this study showed that the combination therapy reduced the number of newly formed vessels. In addition, in the mouse model under the combination therapy, the number of M1 macrophages, natural killer cells, and CD8^+^ cytotoxic lymphocytes increase.^37^ Moreover, in a study by Milanovic et al^45^ with the aim of examining the synergistic effects of DMXAA and Taxol, a single DMXAA injection (27.5 mg/kg) was performed 24h after Taxol (10 mg/kg) injection in mice. DMXAA demonstrated anti-angiogenic and hemorrhagic necrosis of highly vascular U251 glioblastoma xenografts. The selective antivascular effect is attributed to the intratumoral activation of several cytokines including macrophage inflammatory protein 1α, granulocyte-colony-stimulating factor (G-CSF), IL-6, TNF-α,^45^ and interferon-inducible protein 10 (IP-10) chemokine.^46^ Likewise, Rauca et al^47^ used a combination of Simvastatin and DMXAA to elicit potent anti-angiogenic capacity to reduce aggressiveness of B16-F11 melanoma cells in vitro. In this formulation, combined work of two agents provided favorable antioxidant environment to target two prominent molecules involved in melanoma aggression including HIF-1α produced by melanoma cells and arginase-1 produced by tumor-associated macrophages. In a study by Seth et al^48^ the effect of toll-like receptor (TLR) 7/8 agonist nanoparticles (gardiquimod- poly lactide-co-glycolide) combined with DMXAA was investigated. The results showed an improvement of ~ 63% in the survival rate of the tumor model animal receiving the combination therapy. Also, M. Weiss et al^49^ showed that DMXAA, as a ligand for STING, triggers the production of IFN through a series of cascading events and causes tumor regression as the result of successful molecular and cellular immunity involving T lymphocytes and myeloid cells.

DMXAA damages vascular endothelial cells and selectively disrupts tumor blood vessels. Plus, it enhances the effect of the vascular infarction due to an increase in immune cell activity. In previous experiments, a single dose of DMXAA ~20 mg/kg (i.p) in mice is shown to produce a higher therapeutic index with minimum toxicity.^45,46,50^ Accordingly, in our experiments we employed a single dose of 25 mg/kg of DMXAA, which was later coupled with the action of tCoa-NGR in a schedule of two i.v doses administered at 48 h intervals to sustain tCoa-NGR thrombotic effects. Nonetheless, for future studies, the relationship between tumor vascular infarction and immune cell function should be explored to provide insight into the molecular mechanism(s) underlying their synergistic effects. Another consideration is the desired time interval between DMXAA injection and tCoa-NGR to elicit the maximum therapeutic index. As in the case of rapidly growing cancer cells such as murine melanoma models, the interval may not be prolonged. However, for slowly growing cancer models/xenografts a different drug administration schedule should be desired. The last consideration to achieve potent anti-tumor effects is to determine tumor-specific receptor distribution. As the dose of tCoa-NGR depends upon the frequency and distribution of its receptors, integrin, and CD13, in the same way, thrombotic effects and the extent of vascular necrotic infarction in various cancer models can be affected by the dose of the administered coagulating agent.

## Conclusion

Together, the results of this study suggest the superiority of a dual attack on tumor vascular systems, using a VDA to target tumor inner cells and a coagulation factor to target tumor periphery to envision an amplified durable tumor growth inhibition potential.

## Ethical Issues

The experiments conducted strictly in compliance with the regulations of Tabriz University of Medical Sciences, Tabriz, Iran.

## Conflict of Interest

Authors declare no conflict of interest.

## Acknowledgments

The authors would like to thank Tabriz University of Medical Sciences (Thesis number: 93/3-4/3) for funding this work. This study is funded by Tabriz University of Medical Sciences (Thesis number: 93/3-4/3).
